# Butyrate promotes visceral hypersensitivity in IBS model via mast cell-derived DRG neuron lincRNA-01028-PKC-TRPV1 pathway

**DOI:** 10.1128/mbio.01533-24

**Published:** 2024-07-02

**Authors:** Ying-Jie Li, Jing Li, Cong Dai

**Affiliations:** 1 Department of Gastroenterology, First Affiliated Hospital, Jinzhou Medical University, Jinzhou City, Liaoning Province, China; 2 Department of Gastroenterology, First Hospital of China Medical University, Shenyang City, Liaoning Province, China; Johns Hopkins Bloomberg School of Public Health, Baltimore, Maryland, USA

**Keywords:** visceral hypersensitivity, butyrate, short-chain fatty acids, dorsal root ganglion, mast cell

## Abstract

Emerging evidence indicates that gut dysbiosis is involved in the pathogenesis of visceral hypersensitivity (VH). However, how gut microbiota contributes to the development of VH is unknown. Here, we sought to examine the signal transduction pathways from gut to dorsal root ganglion (DRG) responsible for this. Therefore, abdominal withdrawal reflex (AWR) scores, fecal output, fecal water content, and total gastrointestinal transit time (TGITT) were assessed in Con rats, VH rats, rats treated with NaB, and VH rats treated with VSL#3. Fecal microbiota and its metabolite (short-chain fatty acids, SCFAs), mast cell degranulation in colon, lincRNA-01028, miR-143, and protease kinase C (PKC) and TRPV1 expression in DRGs were further detected. VH rats showed an increased fecal water content, a shortened TGITT, an increased abundance of Clostridium *sensu stricto* 1 and increased butyrate in fecal samples, an increased mast cell degranulation, an increased expression of lincRNA-01028, PKC, and TRPV1, and a decreased expression of miR-143 in DRGs compared with control rats, which could be restored by the application of probiotic VSL#3. The above-mentioned detection in rats treated with butyrate was similar to that of VH rats. We further confirm whether butyrate sensitized DRG neurons by a lincRNA-01028, miR-143, and PKC-dependent mechanism via mast cell *in vitro*. In co-cultures, MCs treated with butyrate elicited a higher TRPV1 current, a higher expression of lincRNA-01028, PKC, and a lower expression of miR-143 in DRG neurons, which could be inhibited by a lincRNA-01028 inhibitor. These findings indicate that butyrate promotes visceral hypersensitivity via mast cell-derived DRG neuron lincRNA-01028-PKC-TRPV1 pathway.

**IMPORTANCE:**

Irritable bowel syndrome (IBS), characterized by visceral hypersensitivity, is a common gastrointestinal dysfunction syndrome. Although the gut microbiota plays a role in the pathogenesis and treatment of irritable bowel syndrome (IBS), the possible underlying mechanisms are unclear. Therefore, it is of critical importance to determine the signal transduction pathways from gut to DRG responsible for this *in vitro* and *in vivo* assay. This study demonstrated that butyrate sensitized TRPV1 in DRG neurons via mast cells *in vivo* and *in vitro* by a lincRNA-01028, miR-143, and PKC-dependent mechanism. VH rats similarly showed an increased abundance of Clostridium *sensu stricto* 1, an increased fecal butyrate, an increased mast cell degranulation, and increased expression of TRPV1 compared with control rats, which could be restored by the application of VSL#3. In conclusion, butyrate produced by the altered intestinal microbiota is associated with increased VH.

## INTRODUCTION

Irritable bowel syndrome (IBS) is a common gastrointestinal dysfunction syndrome, which is mainly characterized by repeated abdominal pain with altered bowel habits and should exclude organic diseases that can cause the above symptoms (1, 2). IBS is classified into diarrhea-predominant type (IBS-D), constipation-predominant type (IBS-C), mixed type (IBS-M), and unclassified type (IBS-U). IBS-D is the most common type, characterized by recurrent abdominal pain and diarrhea. The pathogenesis of IBS-D is complex and unclear and is mainly associated with visceral hypersensitivity (VH), abnormal intestinal motility, gut microbiota imbalance, and brain-gut axis abnormality. For example, the Rome IV committee emphasizes that VH and abnormal brain-gut interactions are important pathogenic basis of IBS (3), but the specific mechanisms have not been fully defined.

Gut microbiota imbalance plays a key role in VH and brain-gut interactions. Short-chain fatty acids (SCFAs), including acetic acid, propionic acid, butyric acid, valeric acid, and caproic acid, are produced by bacterial fermentation of dietary fiber. SCFAs exhibit immunomodulatory and anti-inflammatory properties in the pathogenesis of IBS-D (4–6). SCFAs are also important mediators of signal transmission between bacteria and host cells such as intestinal endocrine cells, immune cells, and neurons (5, 7, 8). Currently, some studies have focused on the regulatory mechanism of sodium butyrate in VH. For example, a study by Li et al. found that sodium butyrate enema led to increased visceral sensitivity in rats, and its mechanism may be related to the upregulation of substance *P* (SP) and calcitonin gene-related peptide (CGRP) in dorsal root ganglion (DRG) neurons (9). Another study by Xu et al. also found that sodium butyrate enema induced VH by increased DRG neuronal excitability via activation of MAPK–ERK1/2, which phosphorylates Kv4.2, leading to a reduction in transient potassium current (10). A study by Tsukasa Nozu et al. found that sodium butyrate acted on the AMPK and PPAR-γ pathway to regulate VH (11). Therefore, sodium butyrate can regulate visceral sensitivity through different mechanisms.

Mast cells (MCs) are widely distributed around submucosal vessels and lymphatic vessels, in contact with nerve fibers or adjacent to nerve fibers in the gastrointestinal mucosa (12, 13), and interact with neurons in the gastrointestinal tract. MCs are increased in the intestinal mucosa of IBS patients (13–15). In our previous study, we found that MC degranulation was increased in VH rats. The alteration of intestinal microbiota induced increased MC degranulation and secretion, MC chemotaxis, and adherence by impairing the intestinal barrier in a microbiota-humanized IBS mouse model (16–19), which may contribute to VH.

Large intergenic non-coding ribonucleic acids (linc-RNAs) are a class of non-coding RNA transcripts, which can regulate gene expression by competing for the binding of common microRNAs with protein-coding transcripts and other cellular processes. In a previous study, DRG was confirmed to be responsible for signal transmission in VH. In this study, we further used microarray techniques combined with bioinformatics analysis to identify key differentially expressed RNA related to ion channels, neural excitation, and pain conduction in DRG neuron cells of Con rats and VH rats. We found significant differential expressions of lincRNA-01028 and miR-143 in the two groups of rats. However, the underlying mechanism of lincRNA-01028 and miR-143 in VH is poorly understood.

Transient receptor potential vanilloid 1 (TRPV1) is a membrane protein and a non-selective cation channel that is mainly expressed in primary afferent nerves and nociceptive sensory neurons. TRPV1 is the most important and well-studied nociceptor in VH. The upregulation of TRPV1 in the colonic afferent DRGs may be the target of stress-induced VH in rats (20, 21). Recently, both clinical and animal experiments have shown that TRPV1 is involved in the pathogenesis of VH (20–22). Moreover, our previous studies have demonstrated that TRPV1 expression is increased significantly in DRG neurons, whereas the downregulation of TRPV1 alleviates VH, indicating that TRPV1 plays an important role in the pathogenesis of VH (21). However, the upstream mechanisms by which TRPV1 regulates VH are still unclear.

Protein kinase C (PKC) is a key regulatory enzyme. PKC activation is considered to be the basis for the sensitization of neurons that produce hyperalgesia. PKC is widely distributed in the peripheral and central nervous systems and transmits noxious stimulus signals generated by peripheral nerves to the dorsal horn of the spinal cord to induce and maintain central sensitization. Emerging evidence shows that the PAR2/PKC/TRPV1 pathway is involved in VH (23, 24). In our previous study, we found that the abundance of Clostridiaceae *sensu stricto* 1 and MC degranulation were increased in VH rats (25), MCs and PAR2-TRPV1 pathway were involved in the pathogenesis of VH (21), but the mechanism by which gut microbiota and its metabolites activated DRG neurons was not clear. The purpose of this study was to explore the signal transduction pathways responsible for enhanced neuronal excitability in the DRG and demonstrate that this pathway is responsible for VH *in vitro* and *in vivo* assay.

## MATERIALS AND METHODS

### Animal model

**Fig 1:**
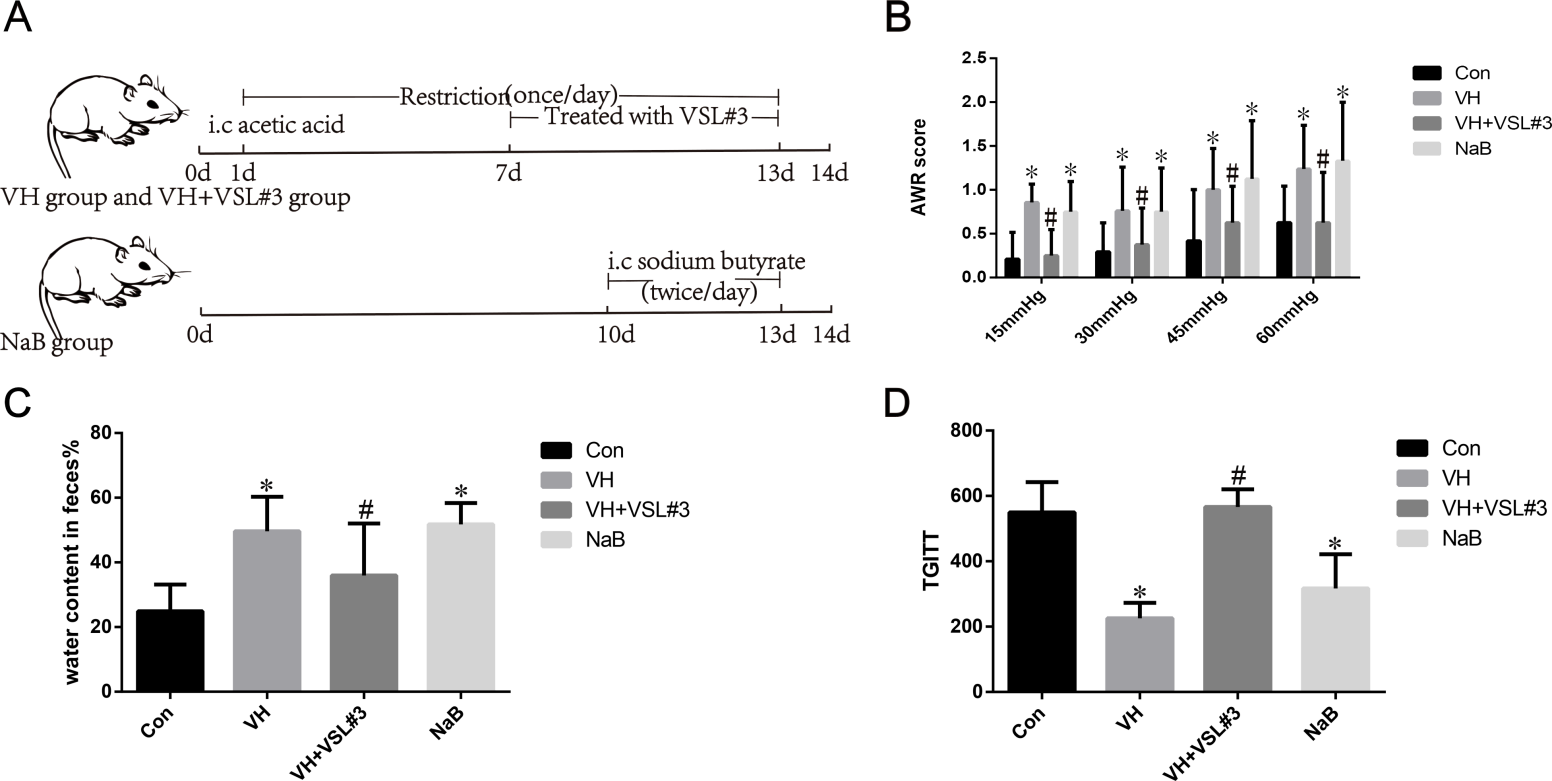
The experiment protocol and comparison of abdominal withdrawal reflex (AWR) scores in different groups. (**A**) The experiment protocol; (**B**) comparison of abdominal withdrawal reflex (AWR) scores in different groups. AWR score in the visceral hypersensitivity (VH) group and NaB group was higher than that in the control group. After administration with VSL#3, AWR scores in VH rats decreased to normal levels. One-way analysis of variance (ANOVA) with least significant difference (LSD) test, *P < 0.05, compared with the control group; ^#^*P* < 0.05, compared with the VH group.

Male SPF Sprague-Dawley (SD) rats (8–9 weeks old, 180–220 g) were maintained as previously described (21). All experiments followed the ethical guidelines for Laboratory Animal Science (Permit No. 2018035). The rats were randomized into four groups (*n* = 8) as described in a previous study (21). All rats were sacrificed on day 14. VH group: briefly, a self-made tube was inserted into the colon through the anus to a distance of 8 cm, and then, 1 mL of 4% acetic acid was injected into the colon through the tube, followed by the injection of 1 mL of phosphate-buffered saline into the colon (26, 27). The rats were fasted for 2 h at the same time each day. VH + VSL#3 group: the VH rats (*n* = 8) received probiotic VSL#3 (Desimone, South Korea, composed of *Lactobacillus paracasei, Lactobacillus plantarum, Lactobacillus delbrueckii subsp. Bulgaricus, Lactobacillus acidophilus, Bifidobacterium breve, Bifidobacterium longum, Streptococcus thermophilus, Bifidobacterium infantis*, 15 mg in 200 µL phosphate-buffered saline (PBS), containing 2.7 billion bacteria) by oral gavage once daily from day 7 to day 13. NaB group: butyrate enema was performed with some modifications (10, 28). Briefly, rats were instilled with 1 mL sodium butyrate solution (110 mg/mL, pH 6.9) into the colon from days 10 to 13, twice daily for 3 days. Control group: untreated rats (Fig. 1).

### Visceral sensitivity evaluation, fecal output, water content in feces, and total gastrointestinal transit time (TGITT)

The abdominal withdrawal reflex (AWR) score was used to assess the visceral sensitivity of rats. The methods were as follows: rats were fasted for 12 hours before the experiment and anesthetized with ether. A silicone catheter with a balloon (8F), connected to a sphygmomanometer and a syringe through a 3-way connector, was inserted into colorectum with a length of 8 cm above the anus, the rats were placed in a specially designed transparent plastic bucket cage (18 × 5 × 7 cm). The rats could only move forward and backward and could not turn around. After complete adaptation (30 minutes), each rat received balloon dilation three times, with a distention of 15 mmHg, 30 mmHg, 45 mmHg, and 60 mmHg, respectively. Each dilation lasted for 5 minutes, with an interval of 30 seconds. AWR score was assessed as follows: 0, no response to colorectal distension; 1, brief head movement followed by immobility; 2, contraction of abdominal muscles; 3, lifting of the abdomen; and 4, lifted pelvis and arched body (21).

The amount of fecal output was collected from 8:00 p.m. on day 13 to 6:00 a.m. on day 14. Weigh the fresh collected feces immediately (wet weight), and then, dry the feces with a hairdryer before weighing (dry weight). Water content in feces = (wet weight of feces - dry weight of feces)/wet weight of feces × 100%.

TGITT was described as the time between intragastric (i.g.) administration of 2 mL of barium sulfate suspension (0.9 g/mL, room temperature) (29) and first white feces excretion on day 13.

### Detection of fecal microbiota and SCFAs

Rats fecal samples (1−5 mg) were collected on day 13 using a sterile Eppendorf tube and immediately frozen at −80°C until processing. 16S ribosomal DNA (rDNA) sequencing of the fecal microbiota and fecal SCFAs was performed at Majorbio Bio-Pharm Technology Co, Ltd (Shanghai, China), as described in a previous study (25).

DNA from the feces was isolated with the E.Z.N.A. soil DNA kit (Omega Bio-tek, Norcross, GA, USA) according to the manufacturer’s instructions. 16S ribosomal DNA sequences (rDNA) were performed using the Illumina MiSeq System (San Diego, CA, USA). The NanoDrop 2000 UV-vis spectrophotometer (Thermo Scientific, Wilmington, DE, USA) was used to determine the final DNA concentration and purification. PCR amplification of the V3-V4 hypervariable regions of the bacteria 16S rDNA gene was carried out using primers 5’-barcode-ACTCCTACGGGAGGCAGCA-3′ and 806R5′-GGACTACHVGGGTWTCTAAT-3′. The resulting PCR products were purified using the AxyPrep DNA Gel Extraction Kit (Axygen Biosciences, Union City, CA, USA) and quantified using QuantiFluor-ST (Promega, Medison, WI, USA). Purified amplicons were pooled in equimolar and paired-end sequenced (2 × 300) on an Illumina MiSeq platform (Illumina). The taxonomy of each 16S rDNA gene sequence was analyzed by the Ribosomal Database Project Classifier algorithm (http://rdp.cme.msu.edu/) against the Silva (SSU123) 16S rDNA database using a confidence threshold of 70%.

The contents of SCFAs in feces were determined by gas chromatography/mass spectrometry (GC-MS). Notably, the fecal sample (50 mg) was mixed with 400 µL of saturated sodium chloride solution and 50 µL of hydrochloric acid saturated sodium chloride solution (3 mmol), followed by shaking, sonicating at low temperature for 1 h, mixed with 500 µL of ether, shaking for 10 minutes. Centrifugation was performed at 12,000 r/min at 4°C for 10 minutes, and the supernatant was collected. Added 0.1 g of anhydrous sodium sulfate to the supernatant and shaken for 3 minutes. After centrifugation at 4,500 r/min at 4°C for 5 minutes, the supernatant was collected and analyzed by GC-MS. Agilent 7890B gas phase and Agilent 5977A mean square displacement (MSD) mass spectrometry were performed under the following conditions: separation was achieved on the Agilent High-Performance Free Fatty Acid Phase (HP-FFAP) capillary column (30.00 m × 0.250 mm × 0.25 µm) with helium (99.9999%) at a constant flow rate of 1.5 mL/min. MSD ChemStation software was used for data processing.

### Histology, electron microscopic for degranulation of MC, immunofluorescence staining and western blot for TRPV1, and quantitative real-time PCR (qPCR) analysis for lincRNA-01028, miR-143, and PKC

The colon specimens were fixed in Carnoy’s fixation; 5-µm sections were processed for hematoxylin-eosin (H&E) staining, and MCs degranulation in colon tissues was detected by electron microscopy (25).

After the examination of the AWR score, rats were euthanized with 10% Chloral Hydrate (0.3 mL/kg) and then decapitated. Bilateral DRGs (L6-S1) were immediately dissected and fused in radioimmunoprecipitation assay (RIPA) buffer. Proteins were quantified by a bicinchoninic acid (BCA) protein assay kit (Thermo). Protein samples of 40 µg from different treatments were loaded onto 10% SDS-PAGE gels and separated by electrophoresis. The proteins were then electrotransferred to a 0.22-µm polyvinylidene difluoride membrane (PVDF; Millipore). The proteins on the blots were then probed by specific primary and secondary antibodies: rabbit anti-TRPV1 (1:1,000, polyclonal, Millipore, UK), rabbit anti-β-actin band intensity (1:2,000, proteintech, UK), and Sheep anti-Rabbit IgG (1:1,500, thermofisher, UK). The bands were identified and analyzed with the Amersham imaging system (Cytiva, USA).

The dissected DRGs were fixed in 4% paraformaldehyde with 30% sucrose at 4°C for 48 h. The fixed DRGs were embedded in Tissue-Tek OCT compound (Leica, Wetzlar, Germany). On a cryostat, the sections were cut in a thickness of 10  µm. The sections were rinsed in PBS and then blocked with 5% bovine serum protein bovine serum albumin (BSA) for 1 h at room temperature (RT). Subsequently, sections were incubated with rabbit anti-TRPV1 antibody (1:500, millipore) overnight at 4°C. After washing with PBS three times on the second day, sections were then probed with the secondary antibodies conjugated with Alexa Fluor 488 goat anti-rabbit IgG (1:500, Invitrogen) for 2  h. The section was analyzed by a confocal laser scanning microscope (IX81-FV1000; Olympus) after being counter-stained with DAPI.

Relative lincRNA-01028, miR-143, and PKC levels in DRGs were measured by qPCR using the ChamQ Universal SYBR qPCR Master Mix (Vazyme) on a QuantStudio3 instrument (Applied Biosystems). Total RNA was extracted using the TRIzol reagent (Vazyme). Reverse transcription was performed by the HiScript II Q RT SuperMix (Vazyme). All primers are shown in Table S1. Every experiment was represented by at least three independent experiments. Gene expression levels were calculated relative to the housekeeping gene 18S using the 2^−△△CT^ method.

### Bioinformatics methods and dual-luciferase reporter assay

The potential miRNA-binding sites of lincRNA-01028 predicted by computer-aided algorithms were obtained from ENCORI, Segal Lab, RegRNA, and microRNA.org-target programs. Wild-type (wt) and mutant (mut) luciferase reporter vectors for lincRNA-01028 or PKC with miR-143 binding regions were constructed by Hanbio Co., Ltd. (Shanghai, China). The vectors were co-transfected with miR-143 mimics into 293T cells, and luciferase activity was measured 48 h later using a dual-luciferase reporter system (Promega, Madison, WI, United States).

### Cell culture

Culture of MCs: MCs were supplied by Cellverse Bioscience Technology Co., Ltd. (Shanghai, China). Bone marrow-derived MCs (BMMCs) from male SD rats were cultured as previously reported (30–33). In brief, bone marrow was cultured in RPMI-1640 medium, supplemented with 10% FBS, 1% penicillin/streptomycin, 10 ng/mL interleukin-3 (IL-3), and 15 ng/mL colony-stimulating factor (CSF). The cell cultures were maintained at 37°C and 5% CO2, changing the medium every 3 days.

Primary culture of rat DRG neurons: DRGs were cultured as described previously (31, 34). In brief, (L6-S1) DRGs were removed from male SD rats (150 ± 200 g) and placed in cold PBS, DRGs were dissected and incubated with collagenase (1 mg/mL) and trypsin (0.25 mg/mL) at 37°C for 20–25 minutes. Washed the cells with 1 mL of culture medium containing fetal bovine serum (FBS) twice, added another 1 mL of culture medium containing FBS, and resuspended the cells in a DRG culture medium. Four milliliters of cell suspension were collected through a sieve of 70 µm filtering out residual tissue blocks in the suspension. The culture medium for DRGs consisted of DMEM: F12, 10% FBS, 0.5% penicillin/streptomycin (Thermo Fischer Scientific), and poly-D-lysine (0.01%) medium. The collected cells were seeded on a 24-well plate, The cell culture was maintained at 37°C and 5% CO2, and the half-well media were replenished every 72 h. DRG neurons were maintained in culture for 6 days (37°C, 5% CO2), then co-cultured it with MCs (31, 34, 35).

Co-culture DRG with MCs on day 7. A transwell chamber was used for co-culture with 10^5^ cells MC in each pore in the superior chamber and DRG in the bottom chamber. MCs were treated with butyrate (5 mmol/L) (36), or DRG neurons were treated with lincRNA-01028 inhibitor (100 mM, RiboBio, Guangzhou, China) prior to MCs treated with butyrate in the co-culture system (6, 37). The TRPV1 channel currents of isolated DRG neurons were recorded using the whole-cell patch clamp technique at room temperature. The expression of lincRNA-01028, miR-143, and PKC in DRG neurons was detected using qPCR.

### Electrophysiological recording

Extracellular solution (Kreb’s) contained (in mM) 140 NaCl, 5 KCl, 2 CaCl2, 10 glucose,1 MgCl2, and 10 HEPES, (pH 7.40, 300–310 mOsm/L). For whole-cell voltage-clamp recordings of TRV1 currents, intracellular solution (Kreb’s) contained (mM) 140 CsCl, 1 MgCl2, 10 HEPES, 5 EGTA, (pH 7.2, adjusted with CSOH, 295–300 mOsm/L).

The TRPV1 channel current on isolated DRG neurons (*n* = 8) was measured by whole cell patch clamp technique at room temperature (23°C−27°C), using Patch Master software (HEKA, Lamprecht, Germany) and EPC-10 patch clamp amplifier. Glass electrode resistance of 2–5 MΩ, with a sampling frequency of 10 kHz and a filtering frequency of 2.9 kHz, was used in patch clamp recording. After obtaining whole cell recording, the holding potential was set to −70 mV, and the effect of MC on capsaicin-induced TRPV1 channel activity was recorded. The size of neurons is 9–20 pF.

### Statistical analysis

Data are expressed as mean ± SD. Statistical comparisons were performed using one-way ANOVA with LSD test, Student’s *t* test. IBM SPSS version 17.0 statistical software (IBM Corp., Armonk, NY, USA) was used for data processing and analysis. PClamp10, GraphPad Prism 5, and Excel were used for data collection, analysis, and processing. Differences were considered statistically significant at *P* < 0.05.

## RESULTS

### Visceral sensitivity, fecal output, fecal water content, and TGITT

AWR score in the VH group (0.86 ± 0.21, 0.76 ± 0.50, 1.00 ± 0.47, 1.24 ± 0.50) and NaB group (0.75 ± 0.35, 0.75 ± 0.50,1.13 ± 0.66, 1.33 ± 0.67) was higher than that in the control group (0.21 ± 0.31, 0.29 ± 0.33,0.42 ± 0.59, 0.63 ± 0.42). AWR score in the VH + VSL#3 group (0.25 ± 0.30, 0.38 ± 0.42,0.63 ± 0.42, 0.63 ± 0.57) was lower than that in the VH group (0.86 ± 0.21, 0.76 ± 0.50,1.00 ± 0.47, 1.24 ± 0.50) (Fig. 1B).

There was no significant difference in fecal output between rats from different groups (Table S2). VH rats showed higher fecal water content and lower TGITT compared with that in Con group (water content in feces%, 49.65 ± 10.65 vs. 25.49 ± 8.25, *P* = 0.000; TGITT, 225.63 ± 47.63 vs. 549.38 ± 93.03, *P* = 0.000), which could be restored by application of VSL#3 (water content in feces%, 35.98 ± 16.04 vs. 49.65 ± 10.65, *P* = 0.019; TGITT, 566.13 ± 54.28 vs. 225.63 ± 47.63, *P* = 0.000) (Fig. 1C and D). Butyrate-treated rats also showed higher fecal water content and shorter TGITT compared with Con group (water content in feces%, 51.74 ± 6.65 vs. 25.49 ± 8.25, *P* = 0.000; TGITT, 317 ± 104.6 vs. 549.38 ± 93.03, *P* = 0.000). Butyrate significantly increased the visceral sensitivity and shortened the time to the first white stool compared with Con group (Fig. 1C and D).

### Fecal microbiota and SCFAs

**Fig 2:**
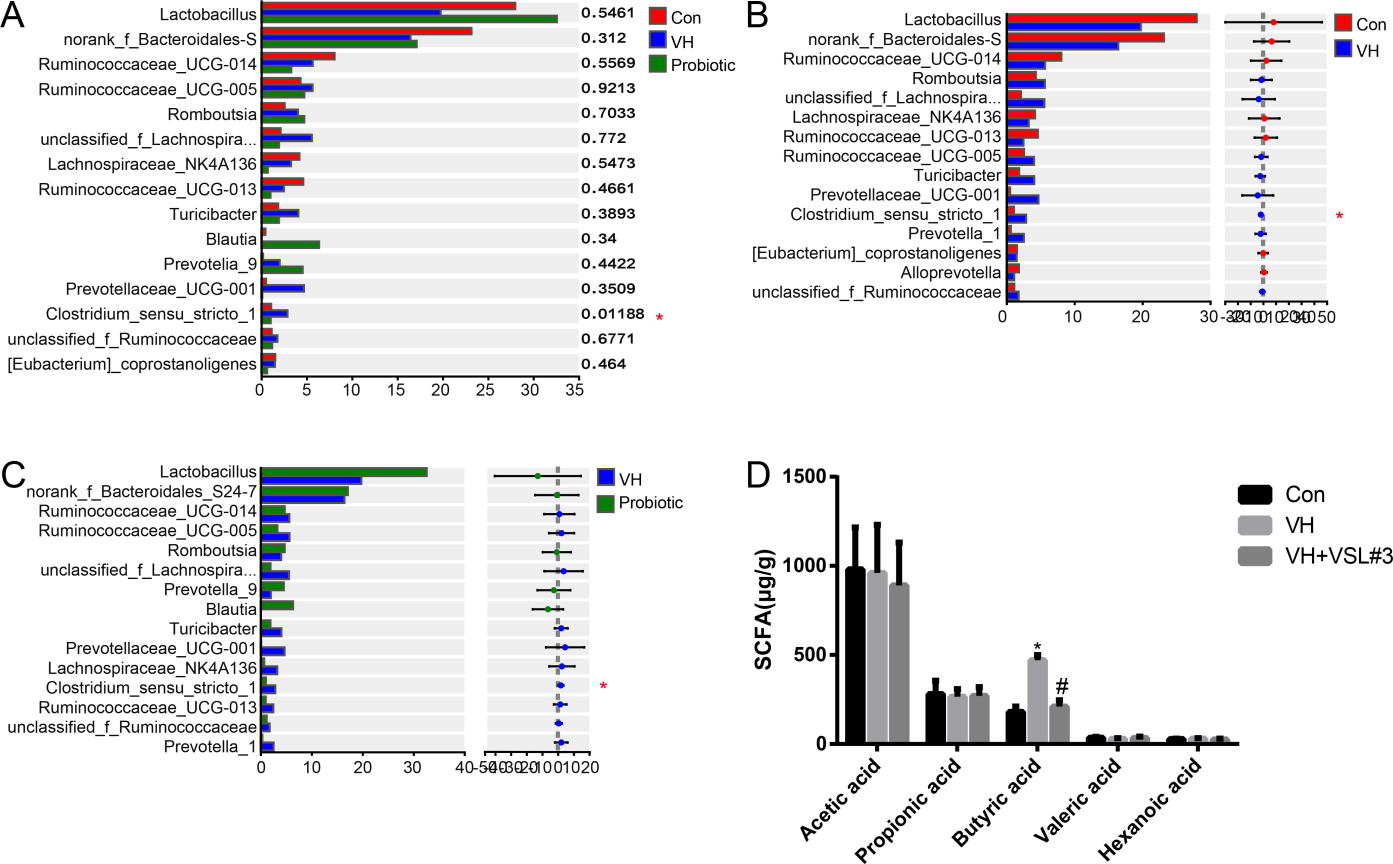
Analysis of fecal microbiota and SCFAs in rats. (**A**) Relative abundance of phylotypes in rats. One-way ANOVA with LSD test, *P < 0.05, compared with the control group; ^#^*P* < 0.05, compared with the VH group. (**B**) Differences in the relative abundance of phylotypes between visceral hypersensitivity (VH) rats and Clostridium *sensu stricto* 1 compared with controls. control rats. VH rats showed a raised abundance of Clostridium *sensu stricto* 1 compared with controls. Student’s *t* test, *P < 0.05. (**C**) Differences in the relative abundance of phylotypes between VH rats and VH + VSL#3 rats. Student’s *t* test, *P < 0.05. (**D**) Fecal levels of acetic acid, propionic acid, valeric acid, hexanoic acid, and butyric acid in different groups. One-way ANOVA with LSD test, *P < 0.05, compared with the control group; ^#^*P* < 0.05, compared with the VH group.

The abundance of Clostridium *sensu stricto* 1 in the VH group was higher than that in the control group at the genus level. VH rats treated with VSL#3 showed a decreased abundance of Clostridium *sensu stricto* 1 (Fig. 2A through C, *P* = 0.01188). No significant difference in other species was found between the groups (*P* > 0.05).

Fecal butyrate levels were higher in the VH rats (470 ± 29 µg/g) than those in the control group (180 ± 30 µg/g). Butyrate levels in the VH + VSL#3 rats (210 ± 35 µg/g) were lower than those in the VH rats (470 ± 29 µg/g) (Fig. 2D). No significant differences in propionate and acetate levels were observed between these groups (Fig. 2D).

### Histology, electron microscopic, immunofluorescence staining for TRPV1, and qPCR analysis for lincRNA-01028, miR-143, and PKC

**Fig 3:**
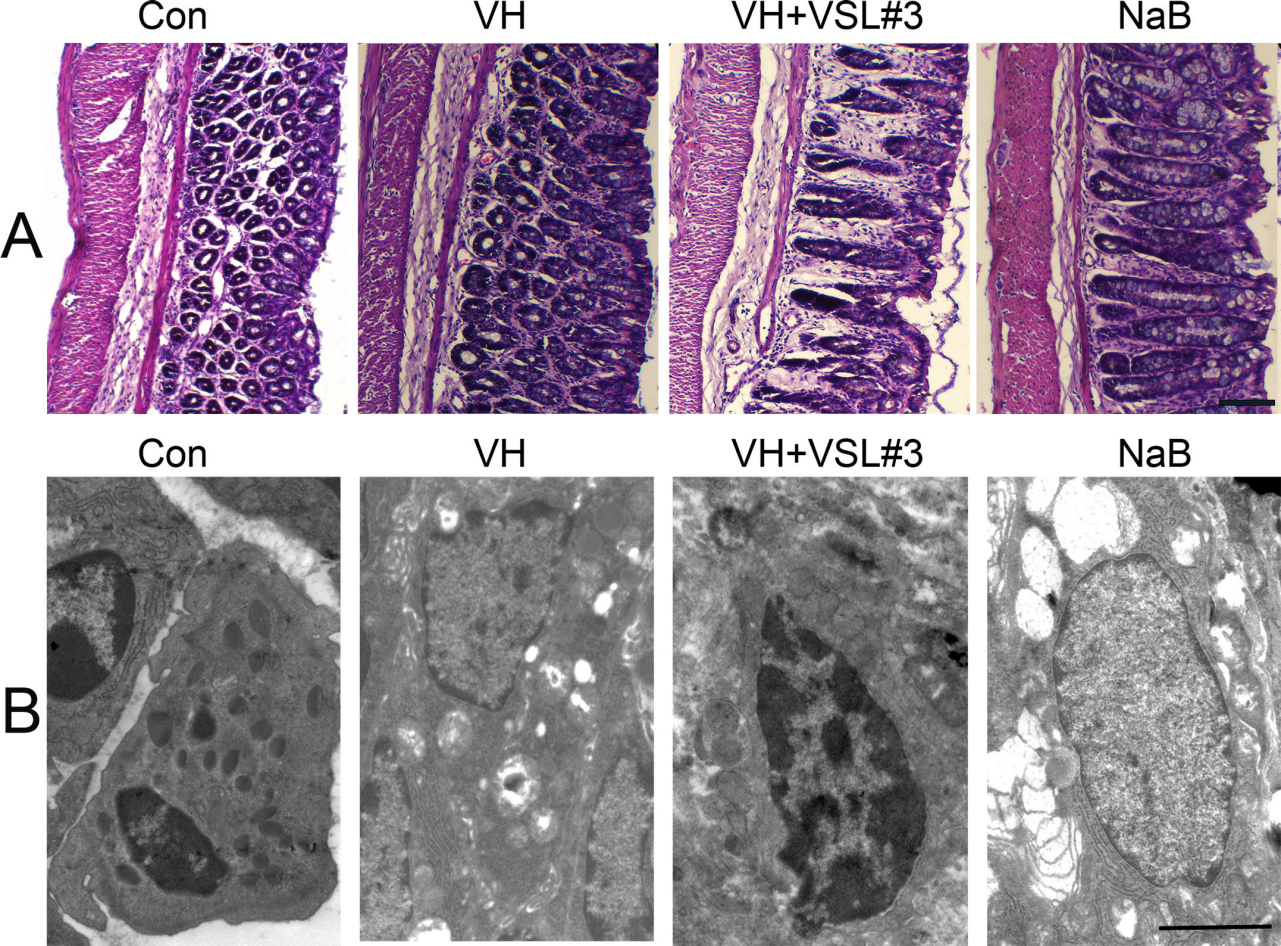
Mast cell degranulation increased in VH rats and NaB rats. (A) Hematoxylin and eosin staining for colon tissue. Scale bar, 200 µm; (B) electron micrograph of mast cell in the rat colon. Scale bar, 2 µm.

**Fig 4:**
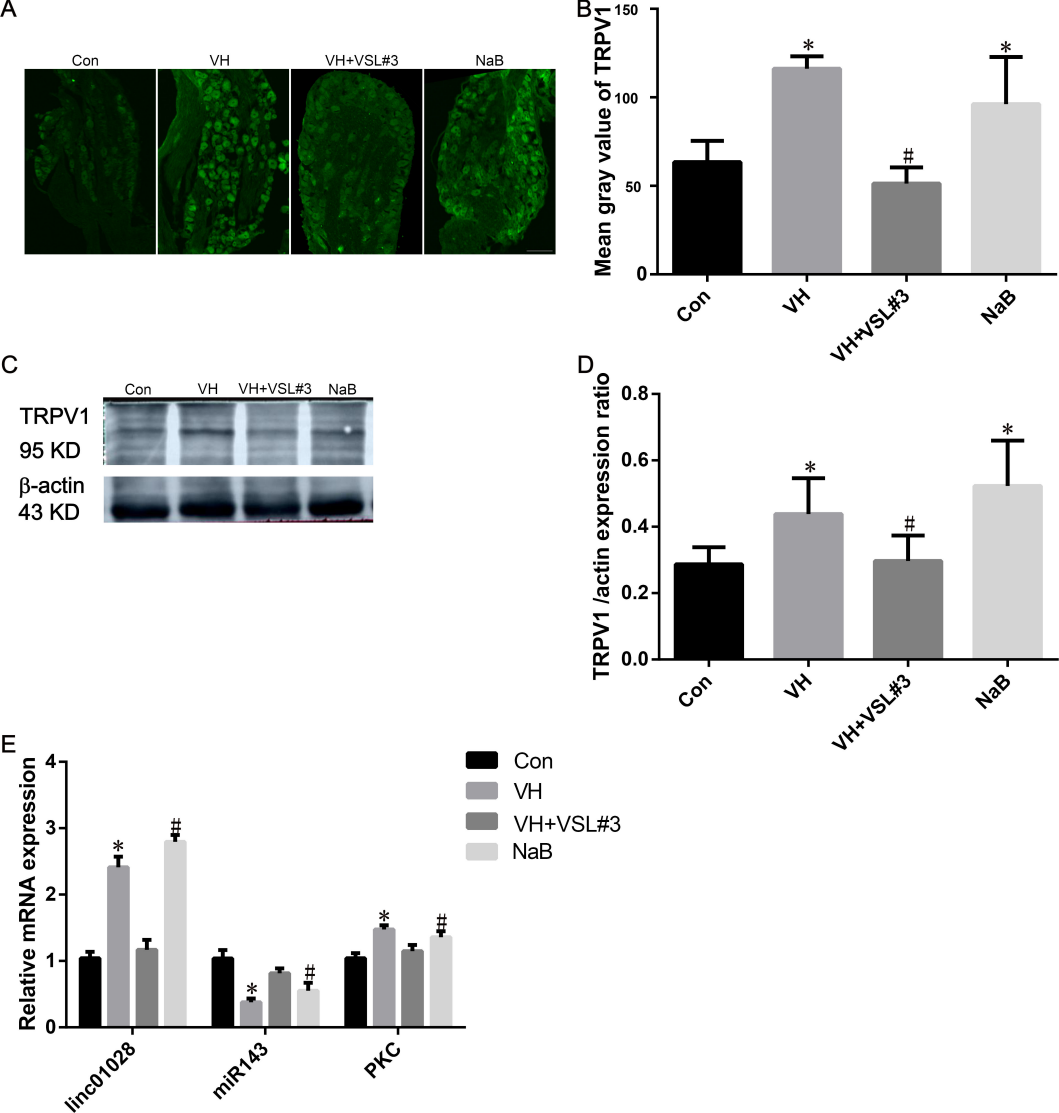
Validation of TRPV1 expression by immunofluorescence and western blot, and linc-01028, miR-143, and PKC expression by qPCR in L6–S1 DRGs of rats. (**A–D**) The expression levels of TRPV1 for validation by immunofluorescence and western blot in L6-S1 DRGs of rats. (**E**) The expression levels of linc-01028, miR-143, and PKC for validation by qPCR in L6–S1 DRGs of rats. Scale bar, 100 µm. One-way ANOVA with LSD test, *P < 0.05, compared with the control group; ^#^*P* < 0.05, compared with the VH group.

In all groups, the colonic mucosal epithelium was intact, and the glands were arranged neatly, with no obvious edema, congestion, inflammatory cell infiltration, or thickening of the muscle (Fig. 3A). The MCs in the control group were regular and round in shape, with bubble-like cytoplasmic particles and intact cell membranes. MCs showed irregular shape, decreased intracellular particulates, and vacuolated cytoplasm in the VH and NaB groups. The degranulation of MCs in VH + VSL#3 rats was lower than that in VH rats (Fig. 3B). The average optical density of TRPV1 in the VH group (0.59 ± 0.17) was higher than that in the control group (0.26 ± 0.07) and VH + VSL#3 group (0.30 ± 0.11) (*P* < 0.05). The average optical density of TRPV1 in the NaB group (0.58 ± 0.12) was higher than that in the control group (0.26 ± 0.07) (Fig. 4A and B). Western blotting indicated that the TRPV1 protein level in the VH group (0.44 ± 0.11) and NaB group (0.58 ± 0.12) in the DRGs was higher than that in the control group (0.29 ± 0.05) (Fig. 4C and D) (*P* < 0.05). The TRPV1 protein level in the VH + VSL#3 group (0.30 ± 0.08) was lower than that in the VH group (0.44 ± 0.11) (*P* < 0.05) (Fig. 4C and D). TPPV1 expression in DRG neurons in the VH and NaB groups was higher than that in the control group. TRPV1 expression in the VH + VSL#3 group was lower than that in the VH group.

The expression of lincRNA-01028 in DRG neurons was increased in the VH group (2.41 ± 2.55) and NaB group (2.79 ± 0.09) compared with the control group (1.04 ± 0.08). The expression of lincRNA-01028 in DRG neurons of the VH + VSL#3 group (1.17 ± 0.12) was lower than that in the VH group (2.41 ± 2.55). The expression of miR-143 in the VH group (0.38 ± 0.05) and NaB group (0.55 ± 0.10) groups was lower than that in the control group (1.04 ± 0.08). The expression of miR-143 in the VH + VSL#3 group (0.81 ± 0.06) was higher than that in the VH group (0.38 ± 0.05). The expression of PKC in DRG neurons was higher in the VH group (1.48 ± 0.05) and NaB group (1.36 ± 0.07) than in the control group (1.04 ± 0.06). PKC expression in DRG neurons of the VH + VSL#3 group (1.15 ± 0.08) was lower than that in VH rats (1.48 ± 0.05) (Fig. 4E).

### CeRNA regulatory mechanism

**Fig 5:**
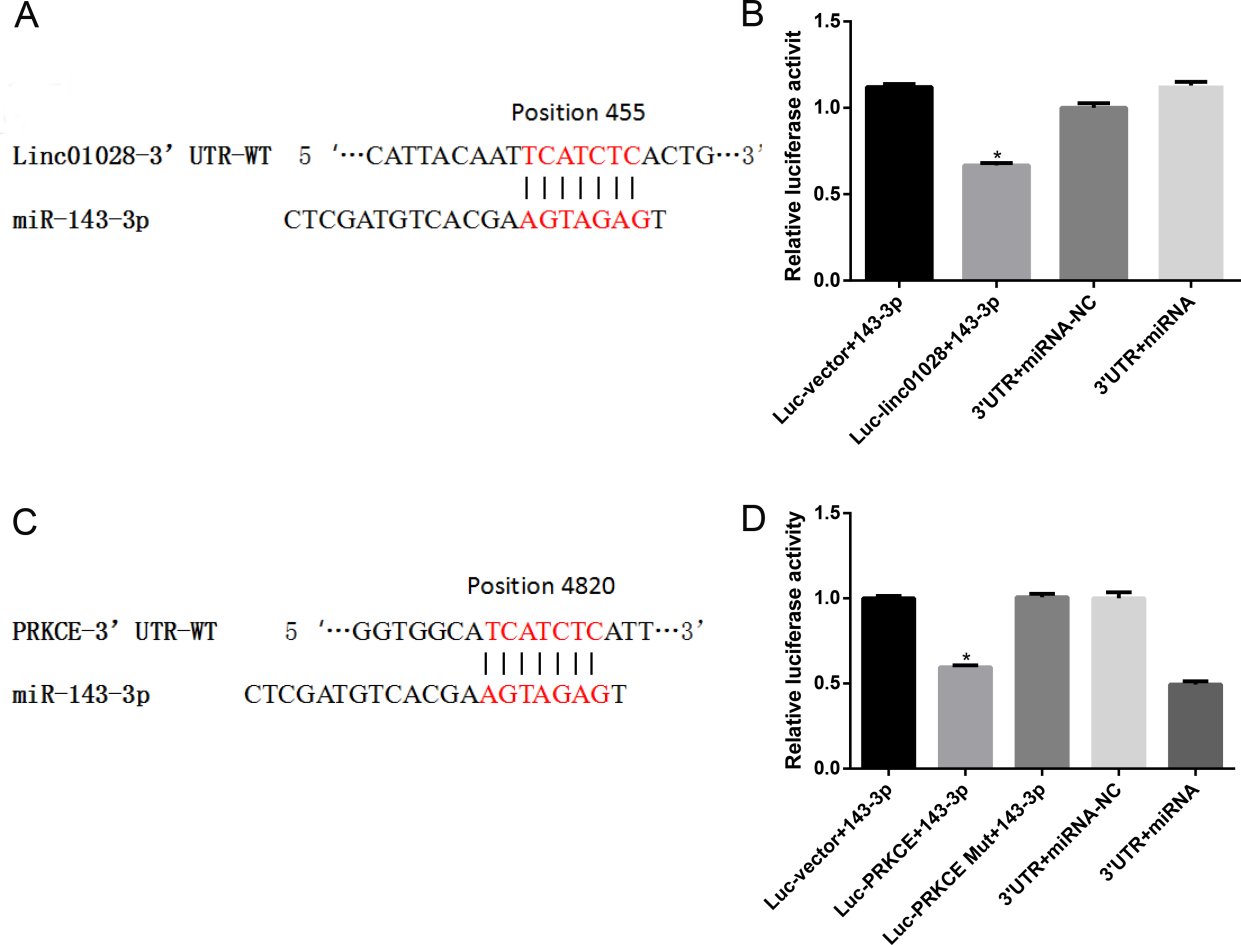
Targeting relationship verified by luciferase report assay. (**A**) Binding sites of linc-01028 targeting miR-143–5p. (**B**) Wildtype binding site sequences of linc-01028 co-transfected with miR-143–5p led to decreased luciferase activity, verifying their targeting relationship. (**C**) Binding sites of PRKCE 3′-UTR targeted by miR-143–5p. (**D**) Wildtype binding site sequences of PRKCE co-transfected with miR-143–5p led to decreased luciferase activity, verifying their targeting relationship. Student’s *t* test.

To investigate the relationship between lincRNA-01028, miR-143, and PKC, we predicted miRNAs using the ENCORI database and identified miR-143 as a possible target of lincRNA-01028. The sequences of wt-lincRNA-01028 and mut-lincRNA-01028 were cloned into luciferase reporter vectors. As shown, the fluorophore activity was reduced in cells co-transfected with miR-143 mimics and wt-lincRNA-01028, whereas no obvious effect was observed in the mut-lincRNA-01028 group (Fig. 5B). PKC is predicted to be a target of miR-143. To confirm this finding, we constructed a luciferase reporter vector with the wild-type or mutated PKC-3′-UTR binding site for miR-143. Reduced luciferase activity was observed in the PKC wild-type reporter (*P* < 0.01) but not in the PKC-mutated reporter (Fig. 5D).

### The expression of lincRNA-01028, miR-143 and PKC, and TRPV1 current in DRG neuron *in vitro*

**Fig 6:**
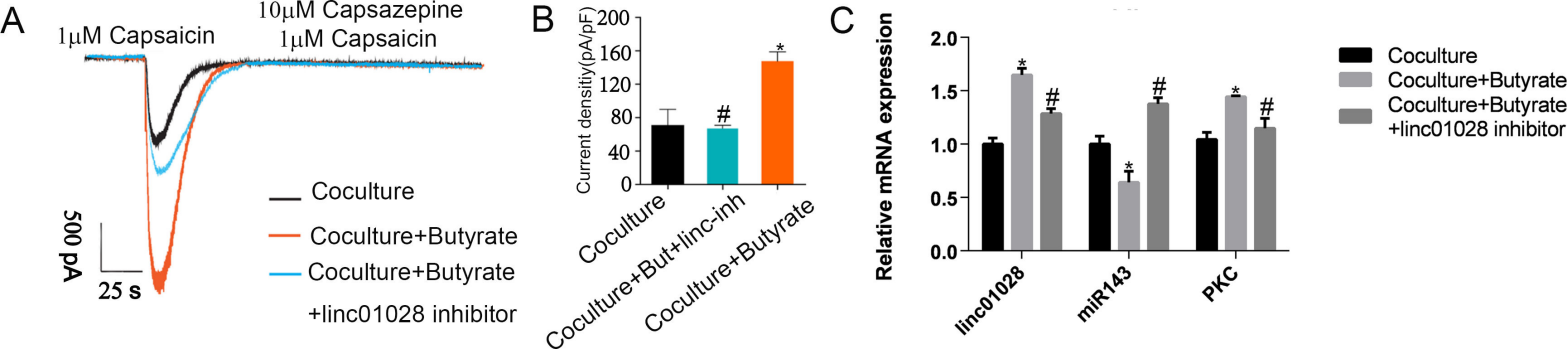
Butyrate modulates TRPV1 sensitization in dorsal root ganglia (DRGs) neurons via lincRNA-01028-PKC pathway. (**A**) Representative current clamp recordings of TRPV1 currents in DRG neurons under different conditions. (**B**) TRPV1 current density analysis. (**C**) Validation of linc-01028, miR-143, and PKC by qPCR. Student’s *t* test, *P < 0.05, compared with the co-culture group; ^#^*P* < 0.05, compared with the co-culture + butyrate group.

The TRPV1 current in the DRG neurons was increased after MCs were treated with sodium butyrate, which could be restored by the application of lincRNA-01028 inhibitor (Fig. 6A and B). We further detected the expression of lincRNA-01028, miR-143, and PKC in the DRG neurons. The results showed that the expression of lincRNA-01028 and PKC was increased, and the expression of miR-143 was decreased in DRG neurons after MCs were treated with sodium butyrate, which could be prevented by the application of the lincRNA-01028 inhibitor (Fig. 6C).

## DISCUSSION

Gut microbiota and its metabolites, such as SCFAs, have been proposed as possible etiological factors for VH (6). VH rats show altered gut microbiota (38–40). The changes in dominant bacteria can change the metabolism level of SCFAs (41). SCFA-producing genera include Clostridium_sensu stricto_1 and Clostridium_sensu_stricto_4. Members of *Clostridium* ss1 exhibit a consistent capacity to synthesize butyrate (42). SCFA is known to exert a variety of physiological effects on the intestine. Increased fecal butyrate levels have been observed in patients with IBS-D, and rectal instillation of butyrate increases visceral sensitivity to colonic distension in rats (43, 44). Rats treated with butyrate enemas exhibited VH. Some studies reported that elevated butyrate levels increased the transit rate in IBS mice, which are involved in the pathogenesis of IBS-D (45). Transcriptome analyses have shown that butyrate induces marked changes in gene expression related to neurotrophic signaling pathways (46). Capsaicin-evoked calcium responses increased in naive DRGs incubated with both sodium butyrate/propionate alone and in colonic supernatants derived from post-inflammatory mice. Microbial-derived SCFA sensitizes nociceptive neurons and may contribute to the pathogenesis of post-inflammatory visceral pain (6, 47, 48). In the present study, we found an increased abundance of Clostridium *sensu stricto* 1 and butyrate in VH rats, which could be restored by application of VSL#3, and butyrate enema-induced VH in rats. In addition, incubation of cultured DRG neurons with butyrate *in vitro* led to sensitization of DRG. These results suggested that gut microbiota and its metabolite butyrate played a role in the pathogenesis of VH.

However, the specific mechanisms of butyrate in VH are still unknown. Two orphan G-protein coupled receptors, GPR41 and GPR43, are discovered to be receptors for SCFA (49, 50). GPR43 is expressed by MCs in the rat intestine, and mucosal MCs can sense SCFA via the GPR43 receptor (51). In our previous and present study, we found both VH rats and butyrate-treated rats showed increased MC number and degranulation, and VH rats also showed increased butyrate, and administration of VSL#3 led to decreased concentrations of butyrate, MC degranulation, and VH. Hence, we speculate SCFAs especially sodium butyrate regulate visceral sensitivity by acting on MCs.

Our previous study showed that the interaction between MCs and DRG neurons led to VH (21). To further explore the corresponding mechanism, microarray techniques combined with bioinformatics analysis were used to detect differential genes related to ion channels, neural excitation, and pain transmission in DRGs from Con rats and VH rats. Finally, lincRNA-01028 and miR-143 were identified. Since emerging evidence suggests that lincRNAs participate in the competitive endogenous RNAs (ceRNA) regulatory circuitry, the ceRNA hypothesis has been proposed. We employed bioinformatics analysis and luciferase assays to validate the direct binding ability of the predicted miRNA response elements to the full-length lincRNA-01028 transcript. As expected, we found that miR-143 could form complementary base pairs with lincRNA-01028. Another key aspect of the ceRNA regulatory network is the identification of miRNA target genes. The downstream target genes of miRNAs and lincRNAs compete to combine with miRNAs to form a regulatory network. Predicted using TargetScan (http://www.targetscan) org/), PKC exists in the binding sites of the miR-143 family, and the binding sites are highly conserved in most species. In this study, qPCR analysis showed that miR-143 expression was inversely correlated with PKC expression in VH rats. LincRNA-01028 and PKC interact with each other through shared miR-143.

Incubation of cultured DRG neurons with butyrate *in vitro* leads to sensitization of TRPV1. Previous studies also confirmed that PAR2-TRPV1 pathway is involved in the pathogenesis of VH. LincRNA-01028 upregulates PKC expression through the ceRNA mechanism in DRG neurons, which in turn leads to the activation and upregulation of TRPV1. We confirmed that the activation and upregulation of TRPV1 led to VH through *in vivo* and *in vitro* experiments using WB, immunofluorescence, and patch clamp techniques.

**Fig 7:**
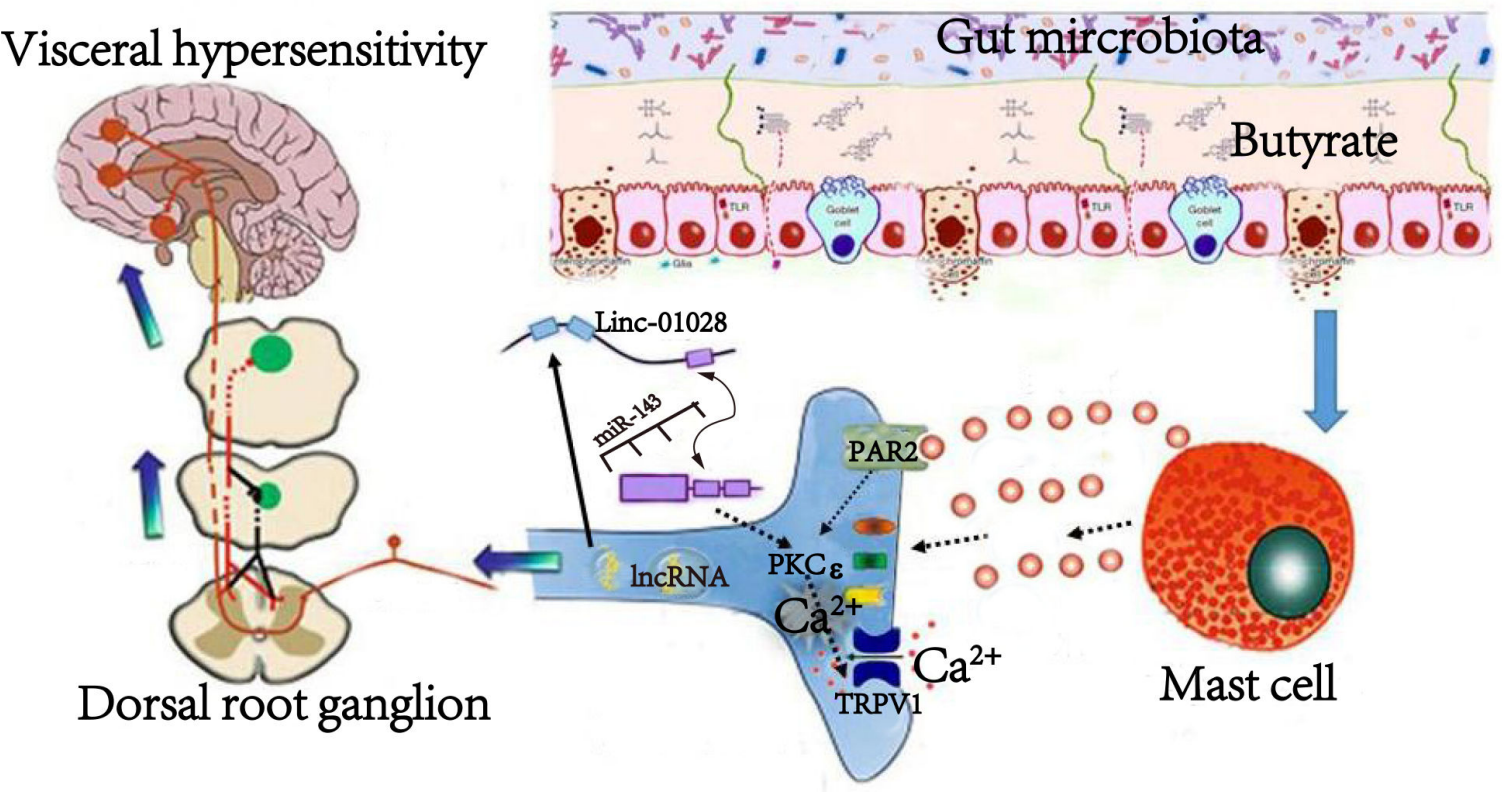
The mechanism of gut microbiota metabolite butyrate-induced mast cells on visceral hypersensitivity by DRG neurons-linc-01028-miR-143-PKC-TRVP1 pathway.

In conclusion, based on the above and our previous research, we found that altered abundance of Clostridium *sensu stricto* 1 accompanied by increased sodium butyrate, which may induce degranulation of MC via GPR receptors, leading to sensitization of DRG neurons. A lincRNA-01028-ceRNA network led to increased PKC, which induced TRPV1 activation and upregulation in DRGs, then induced VH (Fig. 7). The present study identified that lincRNA-01028 was significantly associated with VH. This provides a cellular mechanism to explain the enhanced sensory neurotransmission in butyrate-induced VH and potential therapeutic targets for IBS.

## SUPPLEMENTAL MATERIAL

Table S1 mbio.01533-24-s0001.doc Primers.

Table S2 mbio.01533-24-s0002.doc Fecal output.

ASM does not own the copyrights to Supplemental Material that may be linked to, or accessed through, an article. The authors have granted ASM a non-exclusive, world-wide license to publish the Supplemental Material files. Please contact the corresponding author directly for reuse.
