# Supplementary material for: Butyrate promotes visceral hypersensitivity in IBS model via mast cell-derived DRG neuron lincRNA-01028-PKC-TRPV1 pathway
Source: mBio. 2024 Jul 2;15(8):e01533-24. doi: 10.1128/mbio.01533-24 (PMC11323730; doi:10.1128/mbio.01533-24)
Supplement: Table S1 — Primers. [file mbio.01533-24-s0001.doc]

Table S1 Primer

| **primer** | **Sequences (5′–3′)** |
| --- | --- |
| Linc 01028 | F: GATCGCCGTGTAATTCTAGAAGAGCCACACAGGAAGGAAAAC |
|  | R: CCGGCCGCCCCGACTCTAGATTTTTCACTCATCAGATAACAAC |
| hsa-mir-143 | F: ACGGGCCCTCTAGACTCGAGCCAGAGCTGGAGAGGTGGAG |
|  | R: GTTTAAACTTAAGCTTGGTACCGGAAACACTCTGTCCTTCCTG |
| PRKCE | F: GATCGCCGTGTAATTCTAGACCAGGCCACCTCCTCCCCCTC |
|  | R: CCGGCCGCCCCGACTCTAGATTTCTTTCTTTTTTCTGTTCTTTGCAG |
