# Supplementary material for: Butyrate promotes visceral hypersensitivity in IBS model via mast cell-derived DRG neuron lincRNA-01028-PKC-TRPV1 pathway
Source: mBio. 2024 Jul 2;15(8):e01533-24. doi: 10.1128/mbio.01533-24 (PMC11323730; doi:10.1128/mbio.01533-24)
Supplement: Table S2 — Fecal output. [file mbio.01533-24-s0002.doc]

Table S2 Fecal output

| Group | Fecal output(X±S, g) |
| --- | --- |
| Con | 4.28±1.40 |
| VH | 3.66±1.49 |
| VSL#3 | 4.15±1.22 |
| NaB | 3.87±1.53 |

Con: control; VH: visceral hypersensitivity; NaB: sodium butyrate.
